# Significant difference in cardiac ventricular dimensions when measured using two different standard methods

**DOI:** 10.1007/s12024-023-00579-5

**Published:** 2023-01-27

**Authors:** Jack Garland, Melissa Thompson, Isabella Thompson, Alex Olumbe, Rexson Tse

**Affiliations:** 1 Forensic and Scientific Services, Health Support Queensland, Gold Coast University Hospital, Southport, QLD Australia; 2 Griffith University School of Medicine, Southport, QLD Australia; 3 Faculty of Health Sciences and Medicine, Bond University, Robina, QLD Australia; 4 Department of Molecular Medicine and Pathology, University of Auckland, Auckland, New Zealand

**Keywords:** Postmortem, Autopsy, Heart, Dimensions, Ventricle, Hypertrophy

## Abstract

Cardiac ventricular dimensions measured at postmortem examination are used to assess whether there is hypertrophy of the heart chambers. However, there is no clear consensus on where these measurements should be taken. Some have proposed this should be measured at the mid-ventricular level, but others advocate it should be measured at a set distance (e.g. 20 mm) from the base of the heart. Twenty consecutive adult hearts were examined and showed the ventricular dimensions were significantly higher (mean: 5–15 mm, *p* < 0.01) when measured at a level 20 mm from the base of the heart compared to the mid-ventricular level. Of clinical significance is that in slightly less than half the cases, normal ventricular dimensions at mid ventricle level fell within the criteria considered pathological (> 40 mm) when measured at 20 mm from the base of the heart. In terms of actual ventricular dimensions, only the left ventricle diameter measured at 20 mm from the base of the heart correlated significantly (albeit moderately) with heart weight, suggesting it can be a predictor for cardiac hypertrophy.

## Introduction

Measuring heart dimensions is routine at postmortem examination [1, 2]. These include measuring overall heart dimensions, valve circumferences, ventricle wall thickness, and ventricular dimensions [3]. Ventricular dimensions are useful measurements in assessing cardiac hypertrophy and a dimension of > 40 mm is considered pathological [4]. However, there is no consensus on which level this measurement should be taken at [4]. Some suggest measuring at the mid-ventricular level, whereas some advocating measuring from a standard distance (i.e. 20 mm or 10 mm) away from the base of the heart. This study aims to investigate whether the left and right ventricular dimensions are significantly different at the mid-ventricular level and 20 mm from the base of the heart, and which dimension correlated with heart weight (a marker for cardiac hypertrophy). This would help in standardizing the examination of the heart at postmortem examination.

## Materials and methods

The null hypothesis (H^0^) in the study is that there is no difference in ventricular dimensions when measured at the mid-ventricular level and 20 mm from the base of the heart. The normal ventricular dimensions of both left and right ventricles are between 30 and 40 mm in postmortem examination, and a difference of between 5 and 10% would be considered clinically significant. In relation to the left ventricle, to detect a difference of 3 mm with a standard deviation of 5 mm, a sample size of 18 is needed (*α* = 0.05 and *β* = 0.8). To have a moderate to strong correlation (0.5–0.6), a sample size of 29–19 is needed (*α* = 0.05 and *β* = 0.8). This study subsequently used 20 consecutive cases.

### Case selection

A total of 20 consecutive cases were selected at the Forensic Pathology Department, Gold Coast University Hospital, Queensland. Cases included in the study were adult Caucasian populations where an internal examination was performed as directed by the coroner and had a clear cause of death. Age, sex, body mass index, and causes of death were recorded.

#### Exclusion criteria

- Paediatric population (less than 18 years old).
- Cases with incomplete data set.
- Suspicious and/or homicidal deaths due to potential legal implications.
- Non-Caucasian ethnicity (as documented in the reporting police file), as ethnicity is known to be a confounding factor for heart weight [5–9]. Also, most of the published data and cases admitted are of Caucasian/European descent [10–14].
- Compromised anatomy of the heart (previous surgery, decomposition, and significant trauma), as these would impede the assessment of the heart dimensions and weight.

### Heart examination

The heart was examined in the fresh unfixed state using the short axis method as recommended by the European guideline [3]. The atria and coronary arteries were first examined, and the ventricles were serially sectioned from apex to base in 10 mm intervals up to 20 mm from the base of the heart.

The left ventricular diameter was measured at mid-ventricular level (Lv_mid_) and 20 mm (Lv_20_) from the base of the heart. Similarly, the right ventricle dimensions (a triangular shape) were measured. The anterior–posterior (RvAP) and medial–lateral (RvML) dimensions were measured at the mid-ventricular level (RvAP_mid_ and RvML_mid_) and 20 mm (RvAP_20_ and RvML_20_) from the base of the heart. The trabeculae muscles were discounted when measuring the dimensions. The free walls, interventricular septum, base of the heart, and heart values were examined. After manually removing the blood clots in the heart chambers, the heart was washed/rinsed and pat dried for excess water; the heart was then weighed using a calibrated scale in keeping with recent recommendations [15–17]. The difference between ventricular dimensions (Lv/Rv(AP/ML)_diff_) was calculated (Lv/Rv(AP/ML)_20_–Lv/Rv(AP/ML)_mid_).

**Fig. 1:**
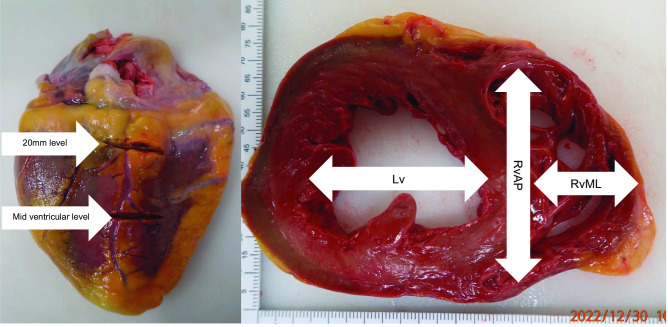
Ventricular dimensions taken at the mid-ventricular level and 20 mm from the base of the heart. The right pane shows how these two levels were established from the posterior aspect of the heart; the left panel shows how the ventricular dimensions were measured

An illustration of where and how these measurements were made is shown in Fig. 1.

### Statistical analysis

Statistical analysis was performed using R 3.6.3 Open source, and a *p*-value of < 0.05 was considered significant. Continuous variables were presented as mean, median, minimum, maximum, and standard deviation. Discrete variables were represented as counts. Student t-test was used to investigate Lv/Rv_diff_ (H^0^: Lv/Rv_diff_ = 0 mm). If there was a difference, further Pearson’s correlation was determined between heart weight ventricular dimensions and Lv/Rv_diff_.

### Ethics approval

This study was approved by the Forensic and Scientific Services Human Ethics Committee (FSS-HEC, HEC 22–20).

## Results

### Case characteristics

In the 20 cases, the mean age was 51.7 years (median: 56, s.d: 15.2, min: 18, max: 73) with a slight male predominance (M:F = 11:9). There were 14 deaths from natural causes, 4 traumatic deaths, and two cases of drug and alcohol toxicity.

The mean heart weight was 391.2 g (median: 354, s.d: 104.3, min: 244, max: 630).

Table 1 shows the cause of death, heart weight, and corresponding ventricular dimensions when measured at the mid-ventricular level and at 20 mm from the base of the heart.

**Table 1:** Cause of death, heart weight (g), and ventricular dimensions (mm) measured at the mid-ventricular level and 20 mm from the base of the heart

Cause of death	Heart weight (g)	Lvmid	Lv20mm	Lv_diff_	RvAPmid	RvAP20	RvAP diff	RvMLmid	RvML20	RvMLdiff
Ischaemic heart disease	244	25	34	9	31	**58**	27	51	71	20
Acute Abdomen	285	25	25	0	42	44	2	22	40	18
Asthma	285	35	37	2	43	52	9	25	**45**	20
Ischaemic heart disease	305	37	40	3	47	50	3	25	37	12
Diabetic ketoacidosis	310	30	35	5	40	**45**	5	40	**45**	5
Acute Abdomen	320	40	**43**	3	42	44	2	30	40	10
Multiple injuries	321	37	**45**	8	33	37	4	23	27	4
Multiple injuries	347	28	40	12	27	**42**	15	35	**50**	15
Pulmonary thromboembolism	350	33	**41**	8	26	**55**	29	19	32	13
Multiple injuries	350	35	40	5	40	**50**	10	50	60	10
Iatrogenic, airway related	358	37	40	3	34	**44**	10	15	**26**	11
Acute abdomen	380	46	53	7	43	49	6	57	60	3
Ischaemic heart disease	400	35	35	0	40	**55**	15	40	**50**	10
Ischaemic heart disease	425	40	**45**	5	50	65	15	35	**55**	20
Drug toxicity	430	30	30	0	50	56	6	20	**50**	30
Multiple injuries	443	35	**47**	12	23	**42**	19	26	**45**	19
Complications from malignancy	515	50	50	0	55	70	15	20	**45**	25
Ischaemic heart disease	530	36	**44**	8	27	**54**	27	35	**55**	20
Complications from malignancy	605	30	**45**	15	30	30	0	25	40	15
Acute Abdomen	630	32	**47**	15	31	40	9	20	39	19

Bolded texts are cases where the ventricular dimensions were normal at the mid-ventricular level and became pathological when measured at 20 mm from the base of the heart; refer to the main text for the abbreviation

### Left ventricle

The mean Lv_diff_ was 6 mm (median: 5, s.d: 4.7, min: 0, max: 15; t-test, *p* < 0.01). Pearson’s correlation coefficient between heart weight with Lv_mid_, Lv_20_, and Lv_diff_ were 0.21 (*p* = 0.38), 0.51 (*p* < 0.05), and 0.45 (*p* < 0.05), respectively. In eight cases, a normal left ventricle diameter fell into the criteria considered to be pathological (> 40 mm) when measured at 20 mm from the base of the heart.

### Right ventricle

#### Anterior-posterior dimensions

The mean RvAP_diff_ was 11.4 mm (median: 9.5, s.d: 8.5, min: 0, max: 29; t-test, *p* < 0.01). Pearson’s correlation coefficients between heart weight with RvAP_mid_, RvAP_20_, and RvAP_diff_ were 0.13 (*p* = 0.57), 0.10 (*p* = 0.68), and 0.03 (*p* = 0.88). In eight cases, a normal right ventricle dimension fell into the criteria considered to be pathological (> 40 mm) when measured at 20 mm form the base of the heart.

#### Medial-lateral dimensions

The mean RvML_diff_ was 15.0 mm (median: 15, s.d: 6.8, min: 3, max: 30; t-test, *p* < 0.01). Pearson’s correlation coefficients between heart weight with RvML_mid_, RvML_20_, and RvAP_diff_ were 0.26 (*p* = 0.26), 0.06 (*p* = 0.80), and 0.35 (*p* = 0.13). In nine cases, a normal right ventricle dimension fell into the criteria considered to be pathological (> 40 mm) when measured at 20 mm form the base of the heart.

## Discussion

The study showed that the left and right ventricular dimensions were clinically and statistically significantly higher when measured 20 mm from the base of the heart when compared to measuring at the mid-ventricular level. This can be explained by the tapering profile of the ventricular chambers from the base towards the apex. In terms of the actual ventricular dimensions, only Lv_20_ correlated moderately and significantly with heart weight. The other ventricular dimensions correlated weakly with no statistical significance with heart weight.

For the left ventricle, the mean difference was 6 mm and up to 15 mm. In four cases, the differences were > 10 mm, and in slightly half (8 out of 20) of the cases, a normal left ventricle diameter became pathological (> 40 mm) when measured at 20 mm form the base of the heart. The differences correlated to the heart weight positively and significantly. This is explained by the increased heart length (distance from base to apex) in heavier hearts rendering an increase in distance from the base to the mid-ventricular level. A similar pattern is seen in the right ventricle with a much higher difference but did not correlate as strongly and significantly with heart weight compared to the left ventricle. The mean difference was > 10 mm and up to 30 mm. Slightly less than half (8–9 out of 20) cases showed a normal right ventricular dimension at mid-ventricular level and became pathological (> 40 mm) when measured at 20 mm from the base of the heart.

In terms of actual ventricular dimensions, only Lv_20_ correlated moderately and significantly with heart weight. This suggests that Lv_20_ has the best predictability for cardiac hypertrophy. This may be because Lv_20_ is closer to the ridged fibrous base of the heart, thus providing a robust width of the heart and being less susceptible to postmortem artifacts such as rigour mortis.

Ventricular dimensions are used to assess whether the ventricle shows any evidence of hypertrophy (concentric hypertrophy or dilatation). Literature and textbooks suggest dimensions of > 40 mm being pathological [4]. However, there is no consensus on where these measurements should be taken [4]. Whilst most textbooks and literature suggest measuring at the mid-ventricular level, others advocate measuring the dimensions at a set distance from the base of the heart [18]. No studies have investigated whether the ventricular dimensions measured are the same between these different approaches. This study showed that when measured at different levels, the ventricular dimensions were statistically different. It is also of clinical significance, as depending on which standard level the measurements were taken, a ventricle dimension can be normal at one level and pathological at another level. Furthermore, this study showed that Lv_20_ was the only measurement that correlates significantly, albeit with moderate strength, with heart weight. This suggests that Lv_20_ would be the best predictor for cardiac hypertrophy and should be used over Lv_mid_ and Rv.

This study was devised to assess the differences in ventricular dimensions when using different standard methods and which measurement correlated with heart weight. It was not designed to develop a conversion method between the two levels or to establish a threshold for cardiac hypertrophy using ventricular dimensions. This study did not investigate whether there is a difference in ventricular walls and interventricular septum thickness at different levels. Thus, further studies would be required to address this.

From the results of this study, it is recommended that when the ventricular dimensions are measured, the exact level should be recorded. Lv_20_ appears to be the most useful predictor for cardiac hypertrophy and is the recommended approach in assessing the heart.

## Key points

1. Cardiac ventricular dimensions can be used to assess cardiac dilation.
2. There is no clear consensus on where these measurements should be taken.
3. This study showed that using different standard methods can yield statistically and clinically different measurements.

## Data Availability

Data for this study can be requested by contacting the corresponding author.
